# A non-transcriptional role for the glucocorticoid receptor in mediating the cell stress response

**DOI:** 10.1038/s41598-017-09722-z

**Published:** 2017-09-21

**Authors:** Marina Ptushkina, Toryn Poolman, Mudassar Iqbal, Mark Ashe, Janni Petersen, Joanna Woodburn, Magnus Rattray, Anthony Whetton, David Ray

**Affiliations:** 1 School of Medical Sciences, Faculty of Biology, Medicine, and Health, University of Manchester, Manchester, M13 9PT UK; 2 School of Health Science, Flinders University, South Australia Sturt Road 5042, GPO Box 2100 Adelaide, Australia; 3 Division of Cancer, School of Medical Sciences, Faculty of Biology, Medicine and Health, University of Manchester, Manchester, M13 9PT UK; 4 Manchester Academic Health Sciences Centre, Manchester, M13 9PT UK; 5 Department of Endocrinology, Manchester Royal Infirmary, Manchester, M13 9WL UK

## Abstract

The glucocorticoid receptor (GR) is essential for the stress response in mammals. We investigated potential non-transcriptional roles of GR in cellular stress response using fission yeast as a model.We surprisingly discovered marked heat stress resistance in yeast ectopically expressing human GR, which required expression of both the N-terminal transactivation domain, and the C-terminal ligand binding domain, but not the DNA-binding domain of the GR. This effect was not affected by GR ligand exposure, and occurred without significant GR nuclear accumulation. Mechanistically, the GR survival effect required Hsp104, and, indeed, GR expression increased Hsp104 expression. Proteomic analysis revealed GR binding to translasome components, including eIF3, a known partner for Sty1, a pattern of protein interaction which we confirmed using yeast two-hybrid studies.Taken together, we find evidence for a novel pathway conferring stress resistance in yeast that can be activated by the human GR, acting by protein-protein mechanisms in the cytoplasm. This suggests that in organisms where GR is natively expressed, GR likely contributes to stress responses through non-transcriptional mechanisms in addition to its well-established transcriptional responses.

## Introduction

The glucocorticoid receptor is a member of the nuclear receptor superfamily, an ancient class of intracellular receptors that has dwindled in number through evolution (NURSA)^1^. In mammals the GR is both integral to the stress response, and also to carbohydrate metabolism. The GR is a ligand activated transcription factor, binding to target sites in the genome as a monomer, or dimer, and has both transactivation, and transrepression activity. Much attention has focussed on the transcriptional regulatory mechanism of GR action, but non-transcriptional roles have also been defined, including regulation of mitotic spindle function^2^. As natural selection in evolution can select for function, and not sequence, we ectopically expressed the human GR in the fission yeast *Schizosaccharomyces pombe (S. pombe)* to test the hypothesis that non-genomic GR signalling is functionally conserved through evolution.

The fission yeast *S. pombe* is a useful model for studying stress response, with conservation of most of the stress-responsive signalling pathways in a single cell organism. In addition *S. pombe* is highly tractable to genetic manipulation. In the past most studies of GR in yeast have used *S. cerevisiae*, in which ectopic GR was shown to be capable of transactivating a transgenic reporter gene^3^. The fission yeast *S. pombe* is evolutionarily divergent from *S. cerevisiae* ^4,5^ *;* in particular the stress-activated MAP kinase pathway is far closer to the mammalian orthologue than is the *S. cerevisiae* ^6^, and GR can serve as an active transcription factor in *S. pombe* if expressed with an appropriate reporter gene, indicating that the GR can be expressed, and retains function^7^.

Accordingly, we sought to identify a non-genomic signalling role for the GR when expressed ectopically in *S. pombe*. We found that ectopic GR conferred heat shock resistant to the *S. pombe*, through a cytoplasmic, non-genomic mechanism. Furthermore, we identified an unexpected role for the translasome^8,9^, with GR interacting with key components in the cytoplasm.

## Results

### Expression human GRα in *S. pombe* cells and identification of a stress tolerant phenotype

**Figure 1:**
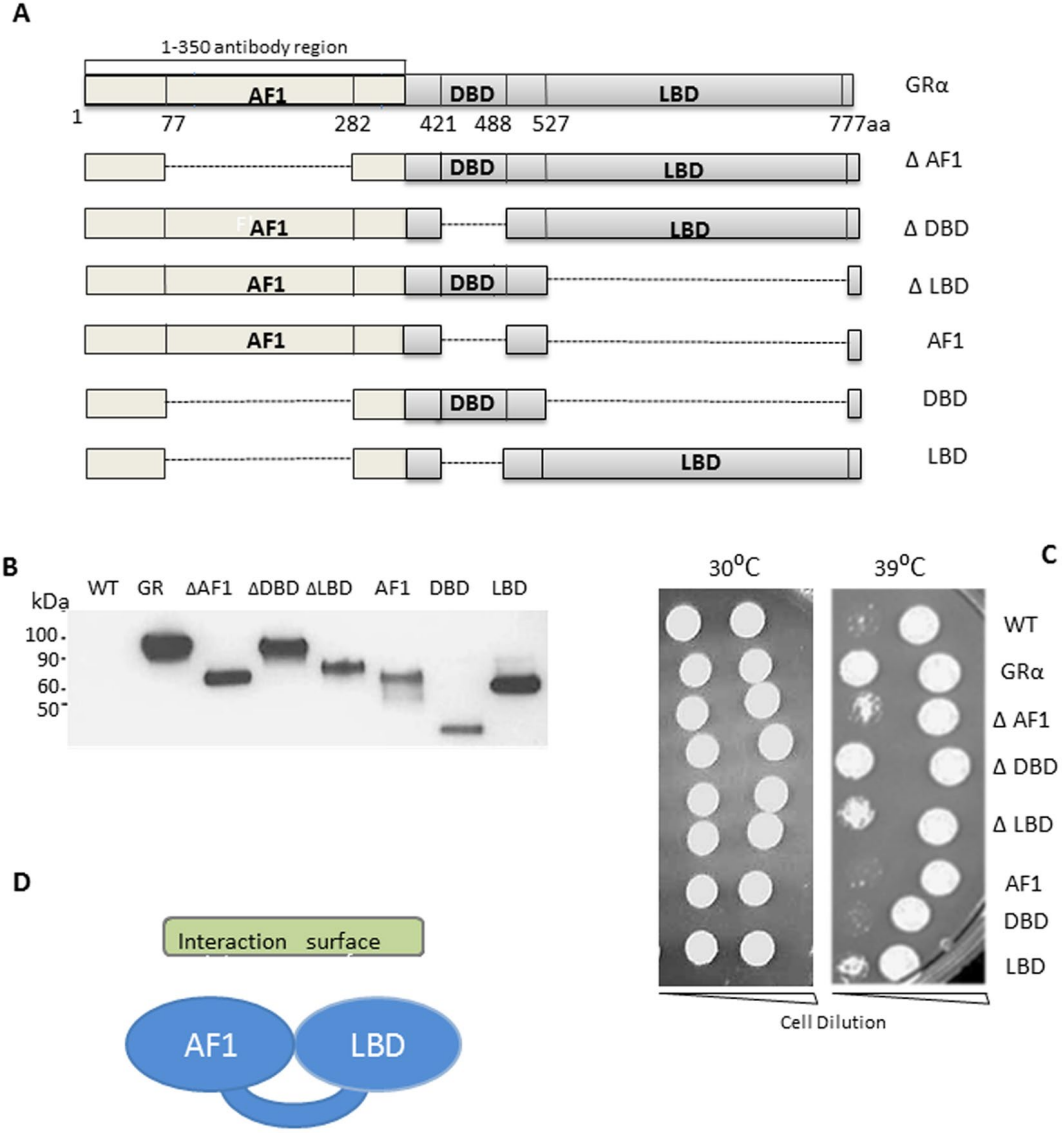
(**A**) Yeast plasmids pRep41/(nmt promoter medium expression, LEU+) and pRep42 (nmt promoter medium expression, Ura+) containing human glucocorticoid receptor sequences (GRα) and the various deletant mutants. (**B**) Western blot analysis of yeast strains transformed with empty vector (WT), or human GR full sequences or disruption mutants. Yeast were grown in EMM selective medium to mid-long phase. Protein lysates were probed for GRα antibody. (**C**) Thermotolerance of transformed yeast. *S. pombe* were transformed with the empty plasmid pRep42 (WT), or with full length human GRα or the disruption mutants as indicated. The transformants were grown in selective EMM. Serially diluted yeast (5 fold dilution series) was spotted on selective minimal media and incubated for 5 days at 30 °C or 39  °C. Results are representative of three independent experiments. (**D**) Summary diagram showing the required N (AF-1) and C (Ligand binding domain, LBD) terminal domains to confer thermoprotection in yeast.

The human GRα was expressed in yeast cells, revealing a single protein species (Fig. 1A,B). Surprisingly, ectopic expression of human GRα conferred a major stress resistant phenotype to the yeast (Fig. 1C). To investigate which GR domain was responsible for conferring this effect a series of deletent GR molecules were expressed in yeast (Fig. 1A,B,C). The deletion mutants conferred variable resistance to thermal stress, with deletion of either the N or the C terminal domains rendering the GR ineffective. However, deletion of the DNA binding domain, required to transmit glucocorticoid signals to target gene enhancers, did not affect thermoprotection (Fig. 1C). Isolated N or C terminal domains alone were insufficient to replicate the protective phenotype (Fig. 1C). This suggests a composite protein surface comprising the two terminal domains in complex is required to signal thermoprotection (Fig. 1D). Although the possibility that WT GR actively regulates genes in *S. pombe* cannot be formally ruled out, the ability of GR variants lacking the DNA binding domain to induce thermoprotection argues that the effect is unlikely to involve direct transcriptional responses.

### Localisation GRα in yeast cells

**Figure 2:**
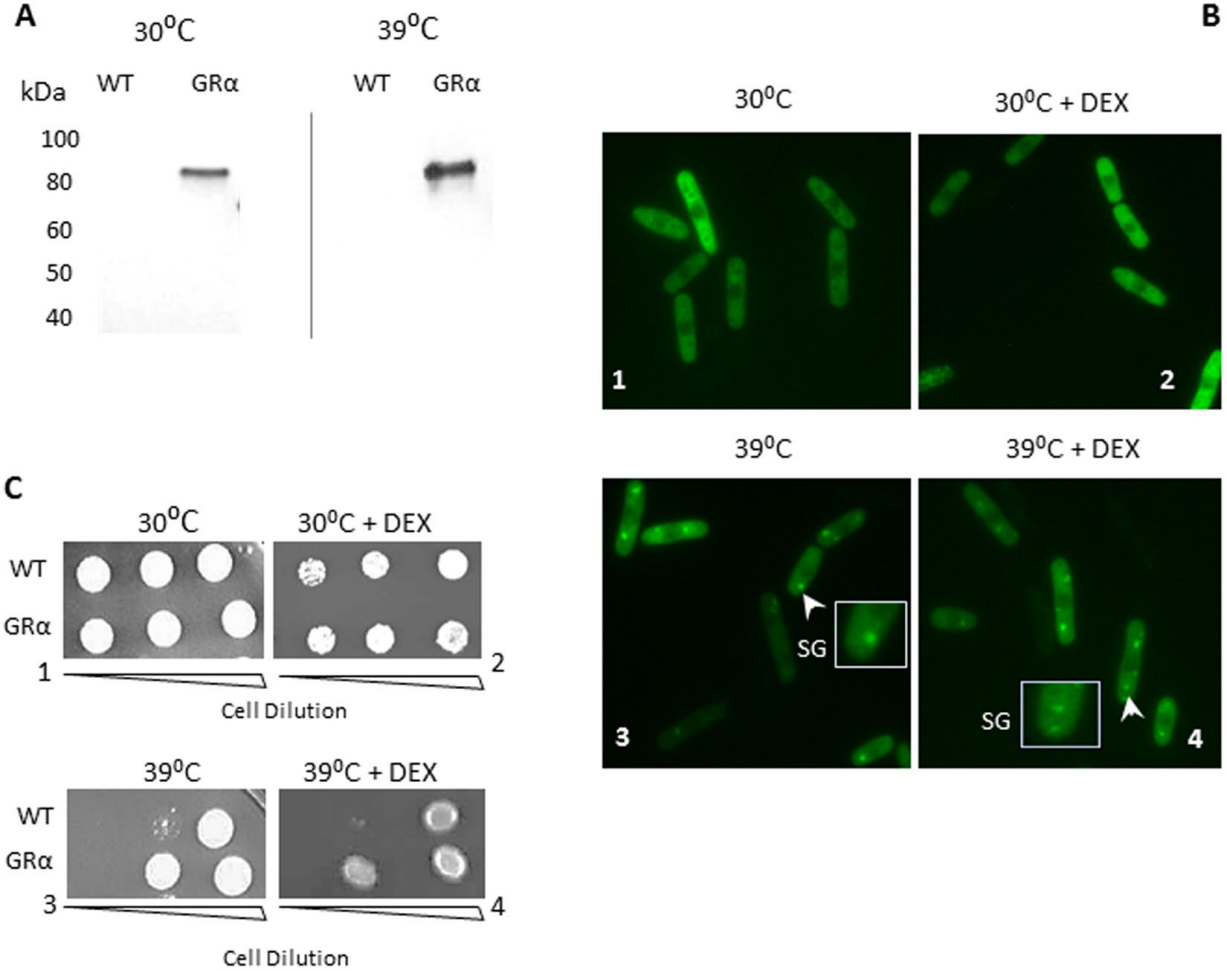
(**A**) Western blot analysis of yeast strains transformed with empty vector (WT), or human GR expression vector (GRα). Yeast were grown in EMM selective medium to mid-long phase. Protein lysates were probed for GRα antibody at 30 °C and at stress temperature 39 °C. (**B**) GRα localisation in yeast cells. *S. pombe* cells were transformed with GRαGFP plasmid. Live cells were examined in different temperatures, and conditions (1) 30 °C and (2) 30 °C+DEX. (3) 39 °C and (4) 39 °C+DEX. The treatment with stress temperature and DEX was for 30 minutes in both cases. Cells were visualized using 100x bright-field objective. Arrows indicate GR containing granules (SG), which emerge at 39 °C. (**C**) Thermotolerance of transformed yeast. *S. pombe* were transformed with empty plasmid pRep42 (WT) or human GRα expressing plasmid (GRα). The transformants were grown in selective EMM medium in present DEX and without DEX. Serially diluted yeast (5 fold dilution series) was spotted on selective minimal media and incubated for 5 days at 30 °C or 39 °C. Results are representative of three independent experiments.

To investigate the localisation, and trafficking, of GRα an N-terminal GFP tag was used. First of all, we checked expression of the GR-fusion proteins under basal and thermostress conditions (Fig. 2A). Human GRα was predominantly cytoplasmic even in the presence of dexamethasone (Fig. 2B2,B4). Thermostress resistance was not affected by ligand activation (Fig. 2C4), in keeping with a lack of nuclear translocation seen with the ligand (Fig. 2B2,4). However, we did observe the accumulation of GRα protein in solid assemblies, likely stress granules (SG), in response to heat shock (Fig. 2B3,4).

### Identification of a protein-protein interaction mechanism conferring stress resistance

**Figure 3:**
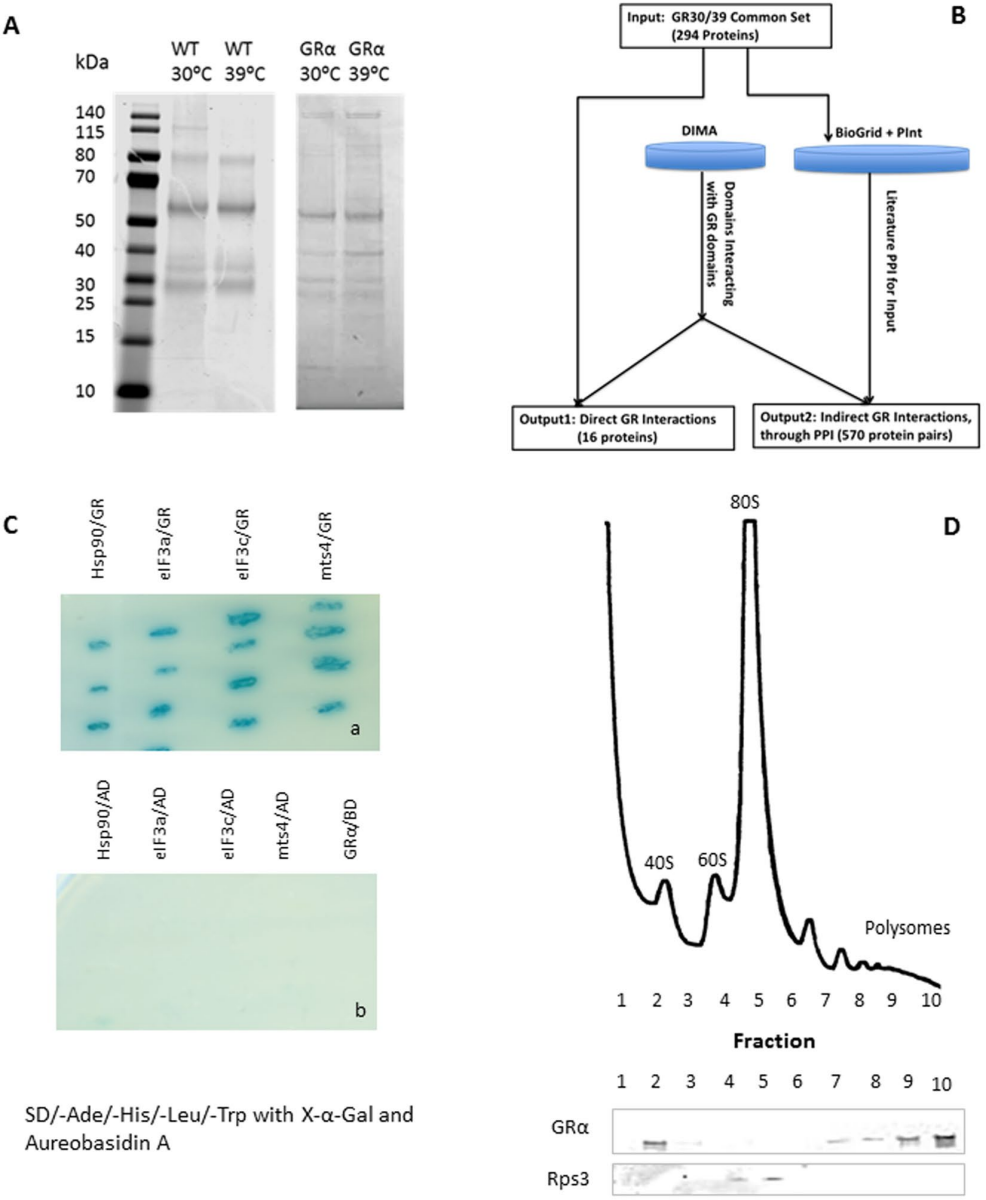
(**A**) Isolation of GRα protein complex in *S. pombe*: WT or GRα strains were subjected to heat shock for 30 min (39 °C), or were grown at 30 °C. Protein complexes were isolated by immunoprecipitation and were subjected to SDS-page. GR interacting proteins were identified by LC-MS/MS (see Material and Methods). (**B**) Schematic diagram showing our computational pipeline (see Material and Methods), taking as input a common set (at both 30 °C and 39 °C temperatures) of GRα co-precipitated proteins, and predicting direct and indirect GRα interactions, as shown in Table 1. BioGrid and PInt are literature sources for *S. pombe* protein-protein interactions, while DIMA is for eukaryotic domain-domain interactions (supplementary Table S1; Materials and Methods). (**C**) Two- hybrid analysis was performed to investigate the binding interaction between GR and the Hsp90, eIF3a, eIF3c, mts4 proteins respectively. An interaction is indicated by activation of Gal-4 responsive promoters for AUR1-C, HIS3, ADE2 and MEL1 reporter genes permitting survival of blue yeast (a)17. The yeast transformants were not able to grow on the same medium if they expressed empty pGADT7 (AD; activation domain) and any of pGBKT7 containing DNA of Hsp90, eIF3 and 19 S genes. Also, yeast did not survive with an empty pGBKT7 (BD; DNA binding domain) and pGADT7 expressing full length human GRα (b). (**D**) Sucrose density gradient profiles of ribosomal material. Yeast extract with GR was fractionated on a sucrose density gradient, fractions were then analysed by Western blotting with anti-GR antibody. Ribosomal protein Rps3 identifies the ribosomal subunit 80 S^43^.

Our identification of a stress-responsive pathway activated by GRα suggested a nongenomic, protein-protein interaction mechanism of action. This was strengthened by the lack of nuclear GRα in either the absence or presence of ligand. To investigate further, we purified GRα-containing complexes using a GRα antibody approach, and subjected the material derived to tandem mass spectroscopy for protein identification. We analysed GRα complexes at both permissive 30 °C, and stress temperatures 39 °C (Fig. 3A). In total 294 proteins common to both temperatures were identified (supplementary Table S1). A gene set analysis using AnGeLi^10^ shows cytoplasmic protein translation as the most significantly enriched GO biological process (33% among these proteins compared to 5% in the background consisting of all protein-coding genes; q-val = 4.06e-58) and cytosol as top localisation (36% as compared to 12% for background; q-val = 4.07e-25) (see the supplementary Table S2).

**Figure 4:**
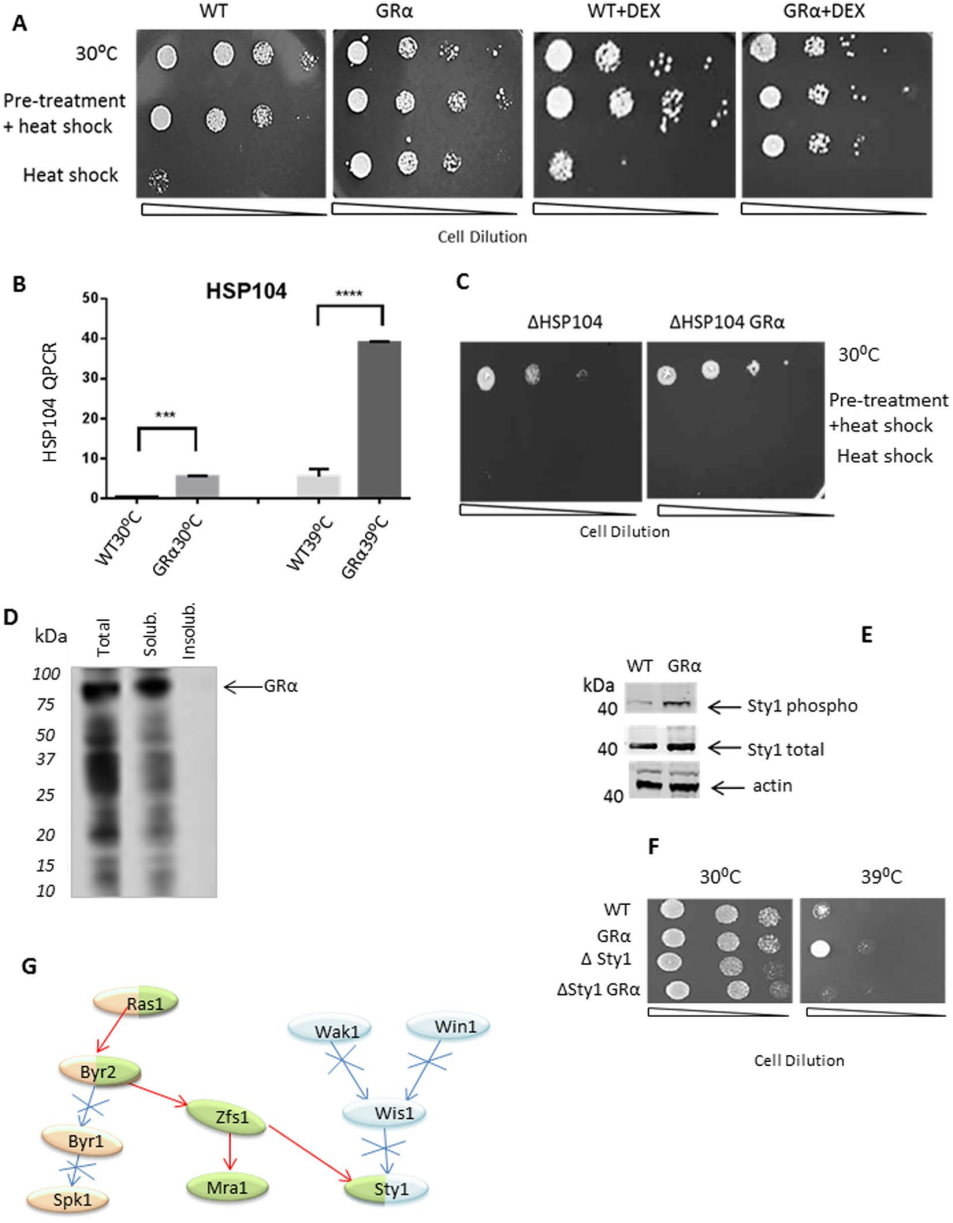
(**A**) Thermotolerance assay. The transformed (URA+) cells, containing empty vector pRep42 (WT) or human GRα expressing vector (GRα) were subject to pre-treatment for 1 h at 37 °C, or not before the heat shock at 50 °C for 20 minutes. Yeast cultures were then serially diluted (serial multiple of ten-fold) before spotting on minimal selective media with or without DEX, and incubated for 5 days at 30 °C. Results are representative of three independent experiments. (**B**) Quantitative RT-PCR for Hsp104 gene. The level of expression of Hsp104 mRNA from control yeast (WT) and yeast expressing human GRα (GRα) was measured by qRT-PCR. The housekeeping gene Act was used as an internal control for gene expression. (**C**) Thermotolerance assay for ∆Hsp104 strain. ∆Hsp104 strain was transformed empty pRep42 plasmid or GRα expressing vector. Transformants were again subject to the thermotolerance assay. (**D**) Insoluble and soluble cytosolic fractions were separated by high-speed centrifugation before the fractions were resolved SDS-Page gel. Western blot analysis shows total, soluble and insoluble fractions probed with anti-GR antibody. (**E**) Western blot analysis of Sty1. Control yeast (WT) and yeast expressing the GRα were grown to an OD595~0.5. Extracts of WT and GRα cells were separated by SDS-PAGE, and probed for total Sty1 using a polyclonal antibody raised to the *S. cerevisiae* Sty1 homolog Hog1. For phosphorylated Sty1 we used the human anti- phospho p38 antibody. Equal loading was measured using an anti Actin antibody. Blots images have been cropped in order to improve the clarity of an effect of phosphorylation. Full images of gels see in Supplementary Fig. 1. (**F**) Sty1 protection from thermal stress. *S. pombe* strain JP198 (∆sty1) was transformed either with a control plasmid, pRep 41, or a human GRα expressing plasmid. Yeast were grown to OD595 ∼ 0.5 and then serially diluted 10 fold, and spotted on minimal media before 5 days at 30 °C or 39 °C. Results are representative of three independent experiments. (**G**) Summary schema of the two major regulatory pathways leading to Sty1 activation: a Ras1 pathway and Wak1/Win1 pathway. Ras1 has been shown to regulate two downstream pathways; one of which is the Byr2/Byr1/Spk1 kinase cascade and the other is the Byr2/Zfs1/Mra1 and Sty1 kinase cascade. Red arrows indicate connections identified within the GR interactome.

The GR interactome at 39 °C contained well-characterised stress granules components (Figs 2B3 and 4, supplementary Table S3), including eIF2, 40 S ribosomal subunit, translation initiation factors^11,12^, also the poly(A)- binding protein and molecular chaperones^13–15^. Two eIF3 subunits, characteristic components of stress granules, were recovered, as well as the prion like protein Hsp104. All three proteins have conserved domains predicted to be capable of forming direct contacts with the GR (Table 1).

**Table 1:** GR co-precipitated proteins with predicted direct GR binding.

ID	Description	Human Ortholog
SPAC926.04c	Hsp90 chaperone	HSP90AA1|HSP90AB1
SPBC16D10.08c	Hsp104 heat shock protein	CLPB
SPBC1734.11	DNAJ domain protein Mas5 chaperone	DNAJA1|DNAJA2|DNAJA4
SPBC1778.01c	zuotin homolog of DNAJ chaperone protein	DNAJC2
SPBC405.06	DNAJ protein Xdj1	NONE
SPAC4A8.16c	translation initiation factor eIF3c	EIF3CL|EIF3C
SPBC17D11.05	translation initiation factor eIF3a	EIF3A
SPBC16C6.07c	19 S proteasome regulatory subunit Rpt1	PSMC2
SPBC4.07c	19 S proteasome regulatory subunit Rpt2	NONE
SPCC576.10c	19 S proteasome regulatory subunit Rpt3	PSMC4
SPBP19A11.03c	19 S proteasome regulatory subunit Mts4	PSMD2
SPAC1565.08	AAA family ATPase involved in ubiquitin-mediated protein degradation Cdc48	VCP
SPCC18.14c	acidic ribosomal protein Rpp0	RPLP0
SPCC285.17	RNA polymerase I upstream activation factor complex subunit Spp27	NONE
SPCC550.14	dbf4,him1,rad35 Hsk1-Dfp1 kinase complex regulatory subunit Dfp1	HDLBP
SPCC576.03c	thioredoxin peroxidase Tpx1	PRDX2

The GR interactome in *S. pombe* was analysed using DIMA as described. The protein ID, description, and human orthologues are listed.

### Computational analysis of GR interactome

In order to analyse these GR interactome data more comprehensively, we developed a computational pipeline which takes as input our GRα-specific interactome and integrates it with the published literature on protein-protein interactions in fission yeast as well as publicly available data on eukaryotic domain-domain interactions in order to predict both direct and indirect binding partners of GRα in *S. pombe* (see Material and Methods).

From the GRα protein interactome (common set of proteins for GRα at 30 °C and at GRα 39 °C supplementary Table S1) we predict 16 direct interactions, i.e., proteins purified with GRα, and having functional domains known to form direct interactions with characterised GRα domains. These direct interacting proteins include Hsp90, and Hsp104, among others, and are shown in Table 1. The GR: hsp90 interaction in particular has previously been very well characterised^16^.

**Figure 5:**
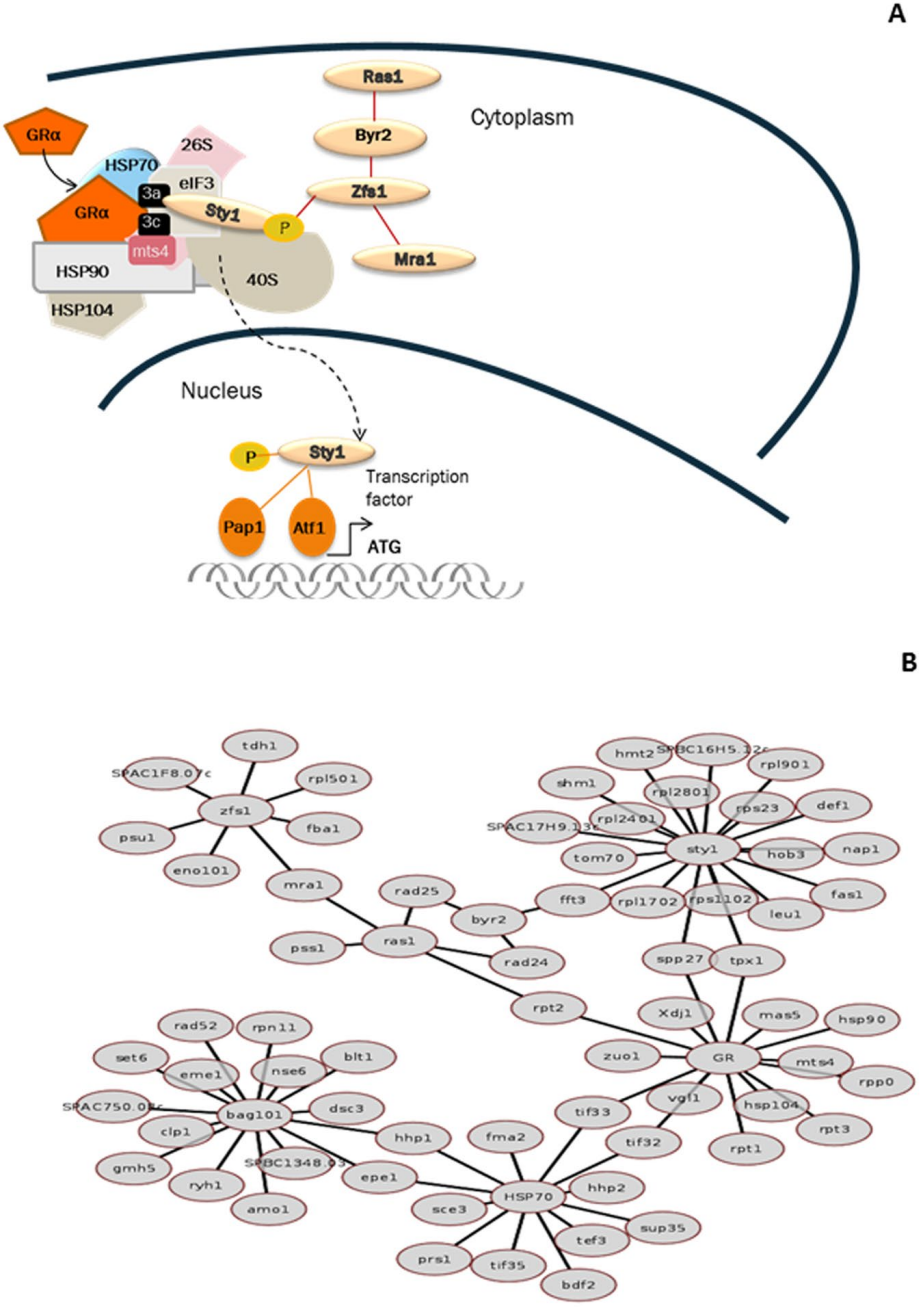
(**A**) Summary diagram indicates the likely site of GR action, lying within a multiprotein complex in the yeast cytoplasm in molecular proximity to the protein synthesis initiation translation factor eIF3, ubiquitin polymerase 26S and ribosomal proteins^8,9^. (**B**) Network visualization of the core GR induced stress response network in *S. pombe*, including direct interactions of GRα with *S. pombe* proteins (in the co-precipitation list), as well as indirect interactions of GRα via other ‘bridge’ proteins (inferred using computational approaches from the co-precipitation list).

Again from the GRα interactome we predict interacting protein pairs where an identified GRα interactor may require a “bridge” protein to explain its presence in the purified complex. These include Ras1, Sty1, and Hsp70 (Fig. 5B). All these 570 predictions and their human orthologues, if available, are given in supplementary table (supplementary Table S4).

### Interaction of GR with eIF3 and proteasome 26S

We selected three from the sixteen genes predicted to make direct contacts with the GR (Table 1) for further analysis. We chose two core subunits of eIF3: eIF3a, eIF3c and one of the 19 S proteasome regulatory subunits, mts4. These were selected based on their presence within the supercomplex translasome that controls protein synthesis and degradation^8,9^, and likely involvement in the stress granule accumulation of the GR observed under conditions of heat stress. In order to prove interaction between GR and these proteins we used an *S. pombe* two hybrid system^17^, using protocols adapted from *S. cerevisiae* ^18^. The gene encoding human GR were fused to the Gal4-activation domain in two-hybrid experiments designed to examine interactions between the genes encoding eIF3a, eIF3c and mts4 were fused to the binding domain of Gal4. As a positive control we used the well-characterised interaction between Hsp90 and the GR^16^, but here we used the *S. pombe* Hsp90. The yeast two-hybrid system revealed that the hGR interacted with all three selected proteins in a robust and ligand-independent manner. The absence of any signal with incomplete hybrid components proves the highly specific nature of these protein interactions (Fig. 3Ca). We checked a number of negative controls including: interaction between the empty pGADT7 and pGBKT7 containing DNA of Hsp90, eIF3a,eIF3c and mts4 genes and interaction between empty pGBKT7 and pGADT7 containing DNA full length human GRα. All these controls were negative (Fig. 3Cb).

### Localisation of GR in polysome gradient

The discovery of GR interaction with components of the protein translasome raised the intriguing possibility that the actions of GR were mediated by direct contacts within that macromolecular complex. In addition to visualising the GR within stress granules, and both finding, and confirming, GR interactions with the eIF3 complex we sought enrichment of the GR within the yeast cell polyribosome fractions. Accordingly, yeast lysates were fractionated through a sucrose gradient, and the fractions analysed for the presence of GR. These studies showed GR emerging in the 40S monomeric ribosome fraction, as well as in the polysome fraction associated with efficient protein translation (Fig. 3D). The eIF3 complex was previously shown to separate in the same 40S ribosomal fraction^19^.

### The role of heat shock protein 104 in mediating GR stress resistance

We proposed the existence of a thermoprotection cascade initiated by the cytoplasmic GR, and resulting in increased expression/activity of a heat-shock protein response, such as the heat shock protein 104 (Hsp104). Therefore, we employed a simple Hsp104 thermotolerance assay^20,21^. As shown in Fig. 4A, human GRα did not affect growth at 30 °C. WT cells do not survive a heat shock of 50 °C for 20 minutes, but pre-treatment at 37 °C for 1 hour before lethal temperature shock resulted in acquired thermotolerance. In contrast, cells expressing human GRα survived a heat shock of 20 minutes at 50 °C in the same way as those cells subjected to pre-treatment at 37 °C for 1 hour. The thermotolerant phenotype conferred by GRα suggested augmented expression of proteins for thermotolerance, such as the GRα interactor Hsp104. Indeed, there was a highly significant increase in Hsp104 gene expression seen with ectopic GRα, both at 30 °C, and at 39 °C (Fig. 4B).

The thermotolerant phenotype was unaffected by ligand activation (Fig. 4A) and, as above, there was no nuclear translocation seen of activated GRα in the *S. pombe* cells (Figs 2B2 and 4). To investigate the requirement of Hsp104 for the GRα thermotolerant effect we used a yeast strain null for the Hsp104 gene^22^. Loss of Hsp104 completely prevented the GRα survival effect (Fig. 4C). Therefore, Hsp104 is required to mediate the thermoresistant phenotype seen with ectopic GR expression, and both under permissive and thermal stress conditions GR expression induce Hsp104 expression, suggesting that a signalling pathway must connect the cytoplasmic GR with Hsp104 induction.

One potential explanation for this is protein misfolding of the GR^23–25^. GR induction of HSP104 may in part be a response to demands placed on the heat shock protein system. This does not appear to be due to overt aggregation of GR (Fig. 4D). However, formation of functional GR complexes is known to involve assembly with chaperone proteins. GR overexpression could thus elicit a compensatory upregulation of the HSP machinery, including HSP104.

### GRα activates stress kinase signalling in *S. pombe*

Hsp104 gene expression is activated by Sty1 kinase^26^. Therefore, we proposed that ectopic GRα activated Sty1, to initiate a signalling cascade leading to Hsp104 expression, so resulting in thermoprotection. Analysis of Sty1 protein revealed no change in expression, but there was a clear increase in Sty1 phosphorylation, and therefore activation, in GRα expressing yeast (Fig. 4E). To investigate the requirement for Sty1 in the phenotype we analysed Sty1 null yeast strain JP198 (see Material and Methods). This yeast grows slowly and all were killed by heat shock, with or without ectopic GRα expression (Fig. 4F). The lack of thermoprotection seen with GRα suggests a critical role for Sty1 in the mechanism of GRα action.

In pursuit of a mechanism linking hGR to Sty1 activation we noted that eIF3a^27^, a known partner of Sty1, and a newly discovered interacting protein with hGR suggested co-location of all three proteins in a cytoplasmic complex, such as the stress granule (Table 1 and Fig. 2B). Now, Sty1 can be activated by either of two pathways: Wak1 or Win1/Wis1/Sty1^6^ and Ras1/Byr2/Zfs1/Sty1 (Fig. 3F)^28,29^. We further interrogated the 294 GR co-precipitated proteins (supplementary Table S1). We discovered a clear enrichment of Ras1, Byr2, Zfs1 and Mra1 as well as Sty1 as present in complex with the hGR (Table 2). This suggests GR engagement with a coherent Ras1/Byr2/Sty1 activation pathway within the cytoplasmic translasome, and stress granule to result in Sty1 activation, and thereby transcriptional activation of Hsp104, and thermoprotection (Fig. 4G).

**Table 2:** Analysis of the Sty1 signalling cascade in *S. pombe*.

Kinases	Interactions from co-precipitation list	# of interactors
Ras1	mts2,pas1,mra1,rad24,rad25	5
Byr2	rad24, rad25,tif3	3
Byr1		0
Spk1		0
Wak1		0
Win1		0
Wis1		0
Sty1	leu1,tpx1,fas1,rpl2401,rpl2801,glu5-kinase, rps23,spp27,hob3,shm1,cfp,hmt2,def1,nap1, tom70,tif3,rpl1702,rps1102,rpl901,hsp104	20
Zfs1	pyr.decab,eno101,tdh1,rpl501,psu1,fba1,mra1	7
Mra1	akt1,zfs1,ras1,gap1, epe1,hhp1,hhp2,pus1, prp43,grs1,mis3,pus4,nop2,brx1,utp22,tRNAguanineN7, dbp3,hrs1,dbl8,nop9	20

Column 1 lists kinases involved in the *S. pombe* Sty1 signalling cascade. Column 2 lists proteins identified as interacting with the GR in co-precipitation studies, and known targets for the respective kinase. The number of identified substrate proteins listed in column 2 is enumerated in column 3.

## Discussion

Nuclear receptors are an ancient family of ligand regulated transcription factors. Their functions have changed through evolution, with the development of sophisticated endocrine signalling cascades feeding into ligand activation of some members, such as the glucocorticoid receptor. Indeed, sophisticated evolutionary biology approaches have delineated the evolution of specific corticosteroid binding through evolution^30^. However, there are inconsistencies in the literature, with some functions ascribed to non-transcriptional mechanisms^31–35^. To investigate these non-conventional mechanisms of glucocorticoid action a tractable model system lacking nuclear GRα was required. We discovered that *S. pombe* supported GRα expression, but not GRα nuclear translocation, thereby offering such a system. In our initial studies we identified acquisition of a striking heat stress resistant phenotype resulting from ectopic GRα expression in *S. pombe*, a discovery which prompted further study.

Human GRα was expressed in *S. pombe*, but there was no detectable nuclear translocation in response to ligand addition. Furthermore, the heat shock resistance was unaffected by incubation with potent GRα ligands, again suggesting a non-transcription mechanism of action. As *S. pombe* and human genomes are evolutionarily divergent conservation of stress regulatory function in both systems by the human GRα is very surprising, and suggests a degree of functional conservation, possibly through preserved protein-protein interactions.

The human GRα interactome in *S. pombe* revealed significant enrichment in chaperone proteins, orthologues of the human heat shock protein partners for GRα. This observation offered a potential explanation for the acquisition of heat shock resistance in the *S. pombe* cells, and indeed we proved the requirement of Hsp104 for mediating ectopic GRα effects.

We observed GRα within cytoplasmic SGs under heat shock in *S. pombe* cells, and analysis of GRα co-precipitated proteins identified the translation initiation components (eIF3a and eIF3c; both direct domain interaction), and the prion-like protein Hsp104 (direct domain interaction). *S. pombe* SGs have previously been shown to contain components of the protein translation machinery including eIF3, eIF2, eIf4G, eIF4A and poly(A)-binding protein, 40 S ribosomal subunits^13^, suggesting that the GR is at least in part located within stress granules, under heat shock. Indeed, we also identify these translated proteins in complex with GR when recovered from heat-shocked yeast, along with well-characterised GR protein chaperones^13–15^. The identification of GRα in SG suggests localisation GR together with translation machinery, a novel mechanism for the nongenomic action in cellular stress response. Interestingly, it was recently shown that the key circadian transcription factor BMAL1 also associates with and regulates the translation machinery, conferring a circadian rhythmicity to protein translation^36^, and another example of a transcription factor acting on translation.

Our interactome studies revealed both direct and indirect GR partners, and inferred the presence of stress-signalling kinases including Sty1, the *S. pombe* orthologue of SAPK^6,37,38^. This prompted us to test the functional role of Sty1 in the GR phenotype, and indeed we were able to show that Sty1 is required to confer a heat resistant cellular phenotype. As we find multiple Sty1 substrate proteins, along with substrates for upstream Sty1 activating kinases, within the GR interactome this suggests the presence of GR within a multi protein complex. This complex includes components of the translasome, within stress granules, where GR can act to activate Sty1, and thereby the expression of Hsp104 to result in thermoresistant yeast. The identification of a cytoplasmic GR mechanism of action in the yeast, which operates through yeast proteins with recognised mammalian orthologues, has major implications for understanding GR action. It is striking that the functional conservation of stress protection from *S. pombe* to mammals operates through a cytoplasmic protein-protein interaction mechanism, and not through direct engagement of the ligand-activated GR with high-affinity genomic binding sites. This discovery offers, for the first time, a coherent explanation for why in mammalian cells the GR occupies a distinct cytoplasmic location in the absence of ligand. This distribution is not seen even with quite closely related steroid receptors, such as the oestrogen receptor. Early explanations suggested that sequestration of quiescent GR away from its binding sites in the genome is important, but if so why does the mechanism not apply more widely across the nuclear receptor superfamily? It is more likely that the cytoplasmic anchoring of GR permits protein-protein interactions to drive non-genomic downstream pathways, as demonstrated in *S. pombe*. However, in mammalian cells distinguishing transcriptional from non-transcriptional mechanisms of GR action has proved more difficult. We now identify not only an important stress-resistant phenotype, but also map a coherent non-nuclear mechanism of GR action.

## Material and Methods

### Strain and plasmids

The *Schizosaccharomyces pombe* strain JP308 (h+ ura 4-D18), JP305 (h+ leu1-32), JP198 (h^−^ styI:: ura4, ∆leu1-32,∆ura4-D18), Bioneer G418 (h+ ∆HSP104:: kanMX4, ade6-M216, ura4-D18,leu1-32) were using in this study. The *Saccharomyces cerevisiae* strain Y2hGold (MATa, trp1-901, leu2-3, 112, ura3-52, his3-200, gal4Δ, gal80Δ, LYS2:: GAL1UAS–Gal1TATA–His3, GAL2UAS–Gal2TATA–Ade2AUR1CMEL1) was used for the two hybrid system (Clontech). Yeast transformations were obtained by the lithium acetate method^39^. Cells were grown in synthetic minimal selective medium.

The human GRα (hGRα) was cloned into Nde1/BamHI site of the pRep41 vector (Leu2) and pRep42 (Ura4) under the control of the medium thiamine-repressible nmt42 promoter^40^. GRα GFP was constructed by GRα N-terminal fusion with green GFP cloned in pRep 42 vector. The GR deletions were generated by excising a BglII/ BglII fragment for the AF1 fragment and by deleting an EcoRI/EcoRI fragment for the LBD, all within the pRep 42 vector. The DBD deletion was constructed by using Site-Directed mutagenesis (Agilent Technologies) to delete between positions 421-488 (Fig. 1A). Vectors pGBKT7 and pGADT7 (Clontech) were used for the two-hybrid system.

### Two‐hybrid protein–protein interaction assay

We adapted *S. cerevisiae* ^18^ two hybrid protocols for application in *S. pombe*. In preparation for two-hybrid *S. pombe* genes Hsp90, eIF3a, eIF3c and mts4 (cDNA collection of Osaka City University) were subcloned in polylinker of vector pGBKT7 (Clontech) containing the DNA-binding domain of the Gal4 translation activator. The gene encoding human GRα was cloned as Nde/BamHI fragment into polylinker region of the pGADT7 AD vector (Clontech) bearing the Gal4-activation domain. After transformation yeast was grown on a leucine‐ and tryptophan‐deficient medium supplemented with X-α-Gal. The blue colonies were analysed by the grown on a leucine‐, tryptophan‐, histidine-, adenine- deficient medium supplemented with the drug Aureobasidin A (200ng/ml) and with X-α-Gal (20 mg/ml) as indicated by the manufacturer (Clontech)^18^.

### Induction and activation of GR

*S. pombe* clones carrying hGRα were growing on selective EMM medium with thiamine concentration 15 µM for repressed nmt-promoter. For activation nmt42 promoter transformants were grown overnight without thiamine. To activate the glucocorticoid receptor we used dexamethasone (DEX) in a final concentration of 10 µM.

### GRα localisation in yeast cells

*S. pombe* strain JP308 were transformed with GFP-green N-terminal fused human GRα pRep42 plasmid. Images were acquired with the camera: http://www.photometrics.com/products/emccdcams/cascade2_1024.php and processed with Metamorph (Universal Imaging).

### Thermoresistance and Thermotolerance assay

#### Thermoresistance assay

The *S. pombe* strain JP308 was transformed with either plasmid pRep42 vector, as control or pRep42 expressing human GRα. Ura + transformants were selected on a plate with minimal selective medium (EMM) supplemented with thiamine. The single colonies were then grown to mid-log-phase in permissive temperature at 30 °C to an OD595~0.5 without thiamine to allow the expression of the proteins. After that a serial dilution of the yeast was prepared (0, 5^−1^, 5^−2^, 5^−3^) in EMM agar minimal medium with or without DEX. Petri dishes were placed at different temperatures (30 °C or 39 °C) and incubated for 5 days.

#### Thermotolerance assay

*S. pombe* strain WT (SP308) and strain ∆HSP104 (G418) were transformed with pRep42 empty plasmid or pRep42 plasmid, expressing human GRα (GRα). Transformed cells were to exponential phase, an OD595~0.5. Cells were pre-treated or not at 37 °C for 1 hour, and then incubated at 50 °C for 20 minutes and cooled on ice for 5 minutes, before being serially diluted (10^−1^, 10^−2^, 10^−3^, 10^−4^), and spotted on the EMM solid media and grown for 5 day at 30 °C.

### Sucrose density gradients

The 80 ml yeast cultures of the strain expressing hGR was grown at 30 °C in EMM medium to an OD_600_~0.6. At this point 2.16 ml chilled formaldehyde was adding. After 1 h, glycine was added (0.1 M final concentration) and the cells were harvested for the preparation of extracts. The extracts were loaded onto 12 ml 15–50% sucrose gradients. Gradients were centrifuged for 2.5 h at 4 °C and 40 000 rpm in a Beckman SW40Ti rotor.

### Protein extraction and immunoprecipitation from *S. pombe*

*S. pombe* cells containing empty vector or vector expressing hGRα were growing in EMM medium without thiamine. Cells were grown to exponential phase (~5 × 10^6^ cells/ml) at 30 °C or 39 °C. Yeast cells were collected by centrifugation for 5 min at 3.500 rpm at RT (room temperature), and washed twice with ice-cold 50 mM Tris.Cl pH 7.5. The pellet was re-suspended in 1 ml cell lysis buffer (250 mM NaCL, 20 mM Tris.CL [pH 7.5], 1% Triton-X100, 100 mM potassium acetate, protease and phosphatase inhibitor cocktail [Roche]) and transferred to Lysing Matrix tubes. The cells were subjected to 5 cycles of 20 seconds, max speed. Supernatants were collected by centrifugation at 13.000 rpm and 4 °C for 5 min. Protein concentrations were determined using the Bradford assay (Thermo Scientific). GRα protein complexes were isolated as previously described^41^. 7ug of anti-GR antibody was bound to 5 mg of epoxy-activated magnetic beads. 2 mL of cell lysate (20 mg/mL) was mixed at 4 °C for 1 hour, before 6 washes with lysis buffer, protein complexes with eluted with lysis buffer containing 1% SDS and heating at 70 °C for 10 min. Eluted material was subjected to SDS-PAGE, gels were stained with Gel Code Blue (Thermo Scientific). Each gel was cut into 6 slices, destained, trypsinized and subjected to LC-MS/MS as previously described^42^. The identification of GRα was also confirmed by mass spectrometry (see supplementary Table S5).

### Western blot analysis

Proteins were separated on SDS polyacrylamide gels were transferred to Pure Nitrocellulose Blotting membrane (Pall). Blots were blocked with 1.2 g milk in 100 ml buffer containing sodium chloride (0.88 g) and 0.1% Tween 20. Membrane was incubated with primary antibody for overnight at 4 °C. Secondary antibody was added to membrane for 1 h at room temperature. Antibodies used in this study: anti-GR (Proteintech), *S. cerevisiae* anti-Hog1 was used to detected total Sty1 (Santa Cruz), human p-p38 antibody (Cell Signalling Technologies) was used to detected p-Sty1, *S. cerevisiae* anti- Rps3 was used in order to identify the ribosomal subunit^43^. Immune complexes were visualized with the Licor Odyssey system.

### Extraction of total RNA, reverse transcription and quantitative PCR

*S. pombe* transformants were grown in selective medium to an OD595 value ∼ 0.5. Cells were collected by centrifuging. The pellet was re-suspended in 100 µl of the following solution: 1 M sorbitol, 0.1 M EDTA (pH 8.0), 0.1% beta-mercaptoethanol, 50 units Zymolase. The cells were incubated at 30 °C for 15–30 minutes, until the sample was clear. After that RNA was purified using the Promega SV Total RNA isolation system according to the manufacturer’s instructions. The resulting RNA was quantified on a Nanodrop ND-1000 spectrophotometer (Labtech International, East Sussex, and U.K.) 1.0 µg of RNA was using for reverse transcribtation (Promega Co-ScriptTM) in a final volume of 20 µl. The RT product was diluted 400 times before use. Quantitative PCR was performed using qPCR kit GoTag qPCR (Promega) on an Applied Biosystems with Optical System software version 2.1. Housekeeping gene Actin was used as an internal control for gene expression. The expression levels of genes of interest are shown as fold change after normalisation to housekeeping gene levels using the normalization against housekeeping gene method.

### High speed subcellular fraction

*S. pombe* cells expressing human GR was grown at 30 °C to OD595~1 and shifted to 39 °C for 1 hour After harvest 4 OD units crude yeast extract was prepared by lysis of yeast cells with glass beads in buffer P (10 mM phosphate buffer [pH 7.5], 250 mM NaCl, 2 mM phenylmethylsulfonyl fluoride and complete cocktail of protease inhibitor (Roche). Cells were broken by vortexing 4 × 30 secs (30 secs vortexing followed 30 secs on ice). Low-speed centrifugation 3 min at 8000 rpm at 4 °C was used to remove cells debris. After that clear extract was fractionated by ultracentrifugation at 100,000 × g for 15 min at 4 °C, TL 100 centrifuge (Beckman). The soluble fraction was recovered and an equal volume of buffer P was added to the pellet fraction. After incubation for 5 min 100 °C in 2 × loading buffer identical volumes of 15 µl soluble and insoluble fractions were used for Western blot analysis.

### Computational Analysis and Expansion of GR-induced interactome in *S. pombe*

Given there is no existing data on GRα interactions with fission yeast proteins, we develop a computational pipeline, to predict potential direct and indirect binding partners of GRα in *S. pombe*. This is depicted as a diagram in Fig. 3B.

Given the input list of proteins (common set of proteins from the precipitation lists at both temperatures), our pipeline works in two ways:

(A) Direct Interactions: We assign domains (PFAM^44^) to the input proteins and using public data on eukaryotic domain-domain interactions (DIMA^45^), we predict direct interactors of GRα, i.e., proteins which have domains which are known to interact with GRα domains.
(B) Indirect Interactions: As there are very few direct interactions, and keeping in mind the effect of potential false positive and false negatives in our experimental data as well as in the public data on protein and domain interactions, we look for potential indirect interactions of input proteins with GRα via other proteins. This is done in two steps given below:

- (B.1) We expand the interactome, using the above list as query to literature PPI resources for *S. pombe*, i.e, BioGrid^46,47^ and Pint^48,49^, keeping only the direct interactions of the proteins in the input list.

- (B.2) Assign the PFAM domains^10^ to the PPIs from (B.1). DIMA is a public resource, which includes experimental as well as computational predictions of domain interactions. From the subset of PPIs in (B.1), we extract those protein pairs, which have at least one domain, which is predicted (in DIMA) to interact with GRα domains.

## Electronic supplementary material

Western blot analyses of Sty1 (full images)

GR interactome in S. pombe.

Gene set analysis of GR interacting proteins.

Protein co-precipitated with GR at 39C

Predicted bridge proteins in S. pombe linking to GR, and their human orthologues.

Identification of GRα peptides by mass spectrometry.
