# Supplementary material for: A non-transcriptional role for the glucocorticoid receptor in mediating the cell stress response
Source: Sci Rep. 2017 Sep 21;7:12101. doi: 10.1038/s41598-017-09722-z (PMC5608759; doi:10.1038/s41598-017-09722-z)
Supplement: Supplementary file 1 — Western blot analyses of Sty1 (full images) [file 41598_2017_9722_MOESM1_ESM.pdf]

A non-transcriptional role for the glucocorticoid receptor in mediating the cell stress response.

Marina Ptushkina[1]*, Toryn Poolman[1], Mudassar Iqbal[1], Mark Ashe[1], Janni Petersen[2], Joanna Woodburn[1], Magnus Rattray[1], Anthony Whetton[3], David Ray[1]**

1 Department of Endocrinology, Manchester Royal Infirmary, CMFT, UK, and Division of Endocrinology, School of Medical Sciences, Faculty of Biology, Medicine, and Health, University of Manchester, Manchester M13 9PT, UK, and Manchester Academic Health Sciences Centre.

2 School of Health Science Flinders University, South Australia Sturt Road 5042 GPO Box 2100

3. Division of Cancer, School of Medical Sciences, Faculty of Biology, Medicine and Health, University of Manchester, Manchester M13 9PT, UK, and Manchester Academic Health Sciences Centre.

* Corresponding author. Tel: + 44 (0) 161 306 0641; E-mail: marina.ptushkina-2@manchester.as.uk

** Corresponding author. Tel: +44 (0)161 275 5655; E-mail: david.w.ray@manchester.ac.uk

Western blot analyses of GRα at 30°C and 39°C (full images)

WT GRα Δ LBD **30° C**

A 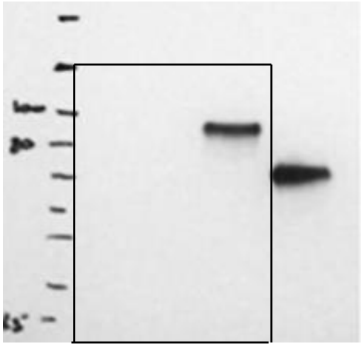

A. Western blot analysis of yeast strains transformed with empty vector (WT), or human GR full sequences (GRα), or disruption mutants (Δ LBD). Yeast were grown in EMM selective medium at 30°C .Black box shows a cropped piece of plot, presented in the article (Fig2.A)

39° C

B 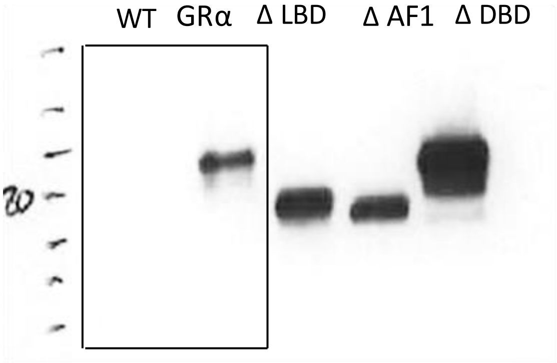

Western blot analysis of yeast strains transformed with empty vector (WT), human GR full sequences (GRα) and disruption mutants: Δ LBD, Δ AF1, Δ DBD. Yeast were grown in EMM selective medium at stress temperature 39°C. Black box shows a cropped piece of plot, presented in the article (Fig2.A)

Western blot analyses of Sty1 (full images)

A

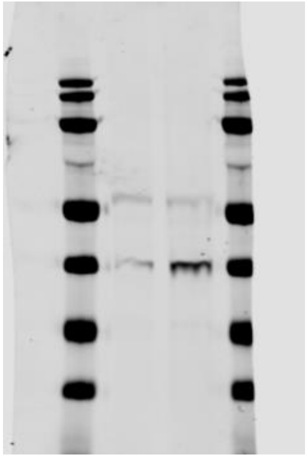

For phosphor Sty1 we are used the human anti- p38 antibody (see Materials and Methods).

B

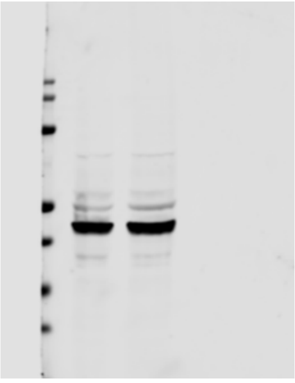

For total Sty1 we are using polyclonal antibody yeast anti- Hog1 (see Materials and Methods).
.

C

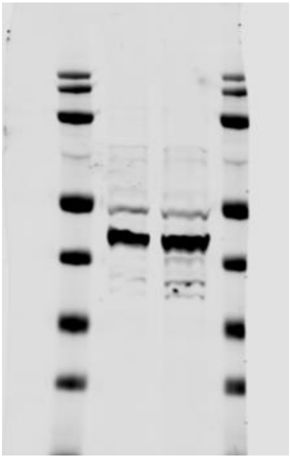

Equal loading was measured using an anti Actin antibody (see Materials and Methods).
